# Structural Diversity of Ultralong CDRH3s in Seven Bovine Antibody Heavy Chains

**DOI:** 10.3389/fimmu.2019.00558

**Published:** 2019-03-22

**Authors:** Jinhui Dong, Jessica A. Finn, Peter A. Larsen, Timothy P. L. Smith, James E. Crowe

**Affiliations:** ^1^Vanderbilt Vaccine Center, Vanderbilt University Medical Center, Nashville, TN, United States; ^2^Department of Pathology, Microbiology and Immunology, Vanderbilt University Medical Center, Nashville, TN, United States; ^3^Agricultural Research Service, United States Department of Agriculture, U. S. Meat Animal Research Center, Clay Center, NE, United States; ^4^Department of Pediatrics, Vanderbilt University Medical Center, Nashville, TN, United States

**Keywords:** lymphocytes, monoclonal antibody, antigen-antibody recognition, *Bos taurus*, ultralong CDRH3, crystal structure, disulfide

## Abstract

Antigen recognition by mammalian antibodies represents the most diverse setting for protein-protein interactions, because antibody variable regions contain exceptionally diverse variable gene repertoires of DNA sequences containing combinatorial, non-templated junctional mutational diversity. Some animals use additional strategies to achieve structural complexity in the antibody combining site, and one of the most interesting of these is the formation of ultralong heavy chain complementarity determining region 3 loops in cattle. Repertoire sequencing studies of bovine antibody heavy chain variable sequences revealed that bovine antibodies can contain heavy chain complementarity determining region 3 (CDRH3) loops with 60 or more amino acids, with complex structures stabilized by multiple disulfide bonds. It is clear that bovine antibodies can achieve long, peculiarly structured CDR3s, but the range of diversity and complexity of those structures is poorly understood. We determined the atomic resolution structure of seven ultralong bovine CDRH3 loops. The studies, combined with five previous structures, reveal a large diversity of cysteine pairing variations, and highly diverse globular domains.

## Introduction

The highly stable immunoglobulin fold is the universal protein scaffold structure for functionally diverse antibodies, enabling mammalian hosts to resist highly diverse pathogens using structures with hypervariable loops displayed on canonical frameworks. The capability for molecular recognition of very diverse antigens stems mainly from the structural diversity of the hypervariable loops of both heavy chains and light chains, which have been termed the complementarity determining regions (CDRs). Because of genetic constraints, principally the limited size of mammalian genomes, all vertebrates except the jawless fish use variable (V), diversity (D), and joining (J) gene segment recombination including non-templated V-D and D-J junctional diversification and heavy/light chain pairing to dramatically diversify the primary antibody repertoire. During antibody maturation, somatic hypermutation mediated by activation-induced cytidine deaminase further expands the diversity of the mammalian antibody repertoire.

Within this general paradigm of antibody diversification, different animals use additional strategies to accomplish structural complexity in antibody repertoires. In chickens or ducks, V(D)J recombination occurs with a single V gene segment, 16 D gene segments, and a single J gene segment. Repeated gene conversion events by upstream pseudogene V segments provide the major mechanism of diversification for V domains of heavy and light chains (1). Structurally, avian antibodies have distinct features compared with those of mice or humans. A sequence analysis of the chicken heavy chain variable domain library revealed that chicken CDRH3s are biased to longer sequences, and they more frequently use small amino acid residues, a higher content of cysteine, and a lower tyrosine content (2). Positions of the cysteine residues suggested non-canonical disulfide bonds within CDRH3s, and between the CDRH3 and CDRH1 or CDRH2 loops (2)(2)(2). Three crystal structures of chicken antibody Fab or single chain variable fragments (scFvs) showed distinct classes of CDRL1 conformations compared with those of antibodies from mice or humans, and the presence of disulfide-constrained, relatively long CDRH3s (3, 4). Similarly, there are limited combinations of V(D)J gene segments resulting from recombination in rabbits. However, rabbits compensate for this limitation with diverse somatic gene conversion events and high levels of somatic hypermutation, thereby making more diverse antibody repertoires than either mice or humans (5). Structurally, rabbit antibodies depend more on light chains for antigen specificity, using longer CDRL3 loops and interdomain disulfide bridges (5). In camelids, B cells produce heavy-chain-only antibodies (HCAbs), in addition to conventional antibodies with heavy/light chain paired configuration. These HCAbs contain some unusual structural features that allow camelids to diversify their antibody repertoires further, including use of various interloop disulfide bonds, an increased surface area of the paratopes, and reshaping of paratopes (6). In the Australian duck-billed platypus, *Ornithorhynchus anatinus*, there is an apparent lack of diversity in germline V segments suggested by a sequence analysis of its heavy chain V regions (7). However, relatively long and highly diversified D segments and N nucleotides compensate well for the germline sequence limitation, diversifying the antibody repertoires. In addition, potential non-canonical disulfide bonds within CDRH3, and between CDRH2 and CDRH3 were suggested because of high probability of presence of cysteine residues in CDRH3 and CDRH2, adding potential structural diversity of the antibodies.

In cattle germline cells, there are only 13 V_H_, 8 D_H_, and 2 J_H_ functional antibody heavy chains, and only one family of V_H_ genes is expressed in bovine antibody repertoires (8). Therefore, diversity created by V(D)J rearrangement in cattle is much more limited than that in mice or humans. A bioinformatic analysis of diversity of antibody heavy chain in four cattle breeds indicated that there are only 162 V(D)J recombination's of germline gene segments that differ significantly (9), compared to the >10,000 possible recombination's of germline gene segments in humans. Under these genetic constraints, cattle have developed a unique mechanism to accomplish antibody diversity by forming the broadest distribution of CDRH3 lengths and the most frequent occurrence of cysteine residues in CDRH3 loops (8–15). The length of bovine CDRH3s features a trimodal distribution: Group 1 comprising very short CDRH3s (≤10 amino acids), Group 2 with intermediate lengths (11 to 47 amino acids) and Group 3 with ultralong CDRH3s (≥48 amino acids). In contrast, human and mouse CDRH3s show a unimodal distribution typically ranging from about 4 to 36 amino acids or 4 to 28 amino acids, respectively (9, 16). Gene segment recombination's encoding group 3 ultralong CDRH3s use the bovine germline D_H_2 gene segment that is 149 nucleotides long. With the mechanisms of V(D)J combinatorial diversity, flexibility in choice of junctional sequences, and a novel nucleotide insertion at the V_H_-D_H_ junction unique to cattle, rearranged bovine V(D)J sequences can encode CDRH3s with lengths up to at least 67 amino acids (17, 18). Additionally, there are 18 RCYW mutation “hotspots” for somatic hypermutation and gene conversion in the bovine D_H_2 gene segment. These genetic peculiarities of bovine B cells greatly enhance the potential for CDRH3 sequence complexity and length. Indeed, previous sequencing of bovine antibody variable domains revealed nearly 10,000 bovine heavy chain sequences with ultralong CDRH3s (18). The sequences were very diverse, but they exhibited conserved cysteines and CDRH3 sequences that clustered to a consensus highly similar to the germline D_H_2 gene segment (18). Furthermore, in addition to the 4 germline-encoded cysteine residues within the D_H_2 gene segment, the biased codon usage in this gene renders point mutations in the DNA exceptionally likely to cause a coding change to cysteine, resulting in even higher high cysteine content in ultralong CDRH3s. The unusual sequence bias for high cysteine content within relatively short sequences can be found in small proteins or protein “minidomains” in all organisms functioning as hormones, growth factors, anti-microbe agents, toxins, enzyme inhibitors (19). These cysteine-rich small proteins or domains usually have distinct three-dimensional structures held together by multiple disulfide bonds instead of being unstructured, for example the knottin fold (19). Therefore, ultralong CDRH3s with greatly diverse sequences and potential multiple disulfide bridges also may form diverse, distinct three-dimensional structures, and the resulting CDRs might provide a unique structural scaffold for diversification of antibody repertoires with possible advantages for antigen recognition.

Recently, five crystal structures of bovine antibody with ultralong CDRH3s were solved (18, 20). Each of the structures showed that the CDRH3s formed an elongated or extended “stalk” and a globular “knob” at the distal ends of the stalks. Knob structures were stabilized primarily by one or multiple disulfide bridges without hydrophobic cores. As expected, the knob structures displayed dissimilar shapes and differing configurations of disulfide bonds, corresponding to the sequence diversity. However, close examination of the structures revealed a few common structural features. Each knob starts with a type I β-turn, and then three antiparallel β-strands connected by loops with different lengths. These extended CDRH3 structures also can be found in human cross-neutralizing anti-HIV antibodies, which use protruding CDRH3 loops to penetrate the HIV glycan shield in order to recognize conserved HIV envelope protein epitopes (21). Remarkably, the capacity of bovine B cells to encode antibodies with protruding CDRH3s has been used to produce cross-neutralizing bovine antibodies with ultralong CDRH3 against HIV envelope glycoprotein by immunizing cows with the soluble cleaved HIV envelope trimer protein BG505 SOSIP, and the knob structure of one antibody plays a dominant role in broad neutralization (22).

The CDRH3s of bovine antibodies offer an opportunity to explore the potential of small globular proteins to achieve extreme structural diversity using limited genetic elements. Here, we solved 7 crystal structures with ultralong CDRH3 structures. The knob structures in these new antibodies revealed four new disulfide configurations and highly diverse shapes. In addition, the knob structures varied greatly, even when the same disulfide configuration was used because of sequence and loop length differences. Remarkably, one of the new structures shows that disulfide bonds can be formed between the knob and the stalk, and the knob structure of this antibody deviates from the common features of the other mAbs discussed above. The highly variable structures of the ultralong CDRH3s seen in this study and in bovine mAbs described by others (18, 20) suggests that the bovine ultralong CDRH3s could adopt numerous different structures reflecting their sequence diversity. This local structural complexity may provide cattle with unique antibody repertoire features to recognize and counteract pathogens. The work also suggests a high potential for using engineered libraries of small globular proteins to achieve structural diversity that could be of wide use in biomedical applications.

## Materials and Methods

### Sequences of Bovine Antibody Heavy Chain Variable Regions

Heavy chain sequences of bovine antibodies with ultralong CDRH3s were obtained from a collection of sequences that we reported previously (23). We chose 33 sequences for recombinant expression based on differing numbers of cysteine residues (2–8 cysteines) and a predicted length (32–65 aa) of the CDRH3.

### Recombinant Fab Expression and Purification

The light chain sequence of the bovine antibody BLV1H12 (PDB ID: 4K3D) (18) was used to pair with the diverse heavy chain sequences in this study. We attempted to express 33 ultralong CDR3 antibodies and were successful in expressing 21 of them as recombinant proteins, ultimately obtaining 7 crystal structures. The wild-type heavy chain sequences for clones BOV-4 and BOV-1 were used for Fab production. Twenty-seven cDNAs encoding recombinant Fab proteins for additional bovine ultralong CDR3s were made by inserting the cDNA sequence for individual CDRH3 sequences into the cDNA background sequence of the clone BOV-2 heavy chain. The genes of light and heavy chains were synthesized (Genscript, Piscataway, NJ), and cloned into a pCDNA3.1(+) vector with CD5 signal peptide, His_6_-tag, and tobacco etch virus (TEV) protease site sequences added at the N-terminus of the expression constructs. Plasmids encoding the light or heavy chains were purified from transformed *E. coli* maxiprep scale cultures (Qiagen, Germantown, MD), and then these DNAs were used to co-transfect the Expi293F cell line (Thermo Fisher Scientific, Grand Island, NY). After 7 days of culture, the medium was separated from cells by centrifugation and filtration. Recombinant Fabs were purified from the medium with a HisTrap Excel nickel affinity column (GE Healthcare, Pittsburgh, PA). After the His_6_-tag was cleaved off by TEV protease treatment (Eton Bioscience, San Diego, CA), Fabs were purified with a second round of chromatography using a nickel affinity column in order to remove uncleaved Fabs, His6-tag, and TEV protease. Finally, size-exclusion chromatography was performed with a Superdex-200 16/60 size exclusion column (GE Healthcare Life Sciences, Pittsburgh, PA) to further purify the samples.

### Crystallization, Data Collection, and Structure Determination

All recombinant Fab protein samples were concentrated to ~12 mg/mL in 20 mM Tris pH 7.5, 50 mM NaCl for crystallization trials. The crystallization and cryo-protection conditions for the Fabs are shown in Table S1. Protein crystals were flash-frozen in liquid nitrogen after a quick soaking in the corresponding cryo-protection solution. Diffraction data were collected at the Beamline 21-ID-G at the Advanced Photon Source, Argonne National Laboratory. The diffraction data were processed with XDS (24) and CCP4 suite (25). The crystal structures were solved by molecular replacement using the crystal structure of bovine antibody BLV1H12 (PDB ID 4K3D) as the initial searching model with the program Phaser (26). The structures were refined and rebuilt manually with Phenix (27) and Coot (28), respectively. PyMOL was used to make all of the structural figures (29). The data collection and refinement statistics for all the crystals is shown in Table S2.

## Results

### Overall Structures of Heavy Chain Variable Domains of the Bovine Antibodies

Here, we sought to further explore the structural diversity of the ultralong CDRH3s of bovine antibodies. We used a collection of bovine antibody heavy chain variable gene sequences we had obtained previously from the blood of domestic cows (*Bos taurus*) using single-molecule long-read sequencing techniques (23). We analyzed the sequences to identify those that were predicted to encode ultralong CDRH3 regions. We chose 33 different bovine heavy chain sequences with ultralong CDRH3s based on differing numbers of cysteine residues (2–8 cysteines) and length (32–65 amino acids) of CDRH3. From 21 expressed constructs, we were able to express Fabs and solve the crystal structures for 7.

**Figure 1:**
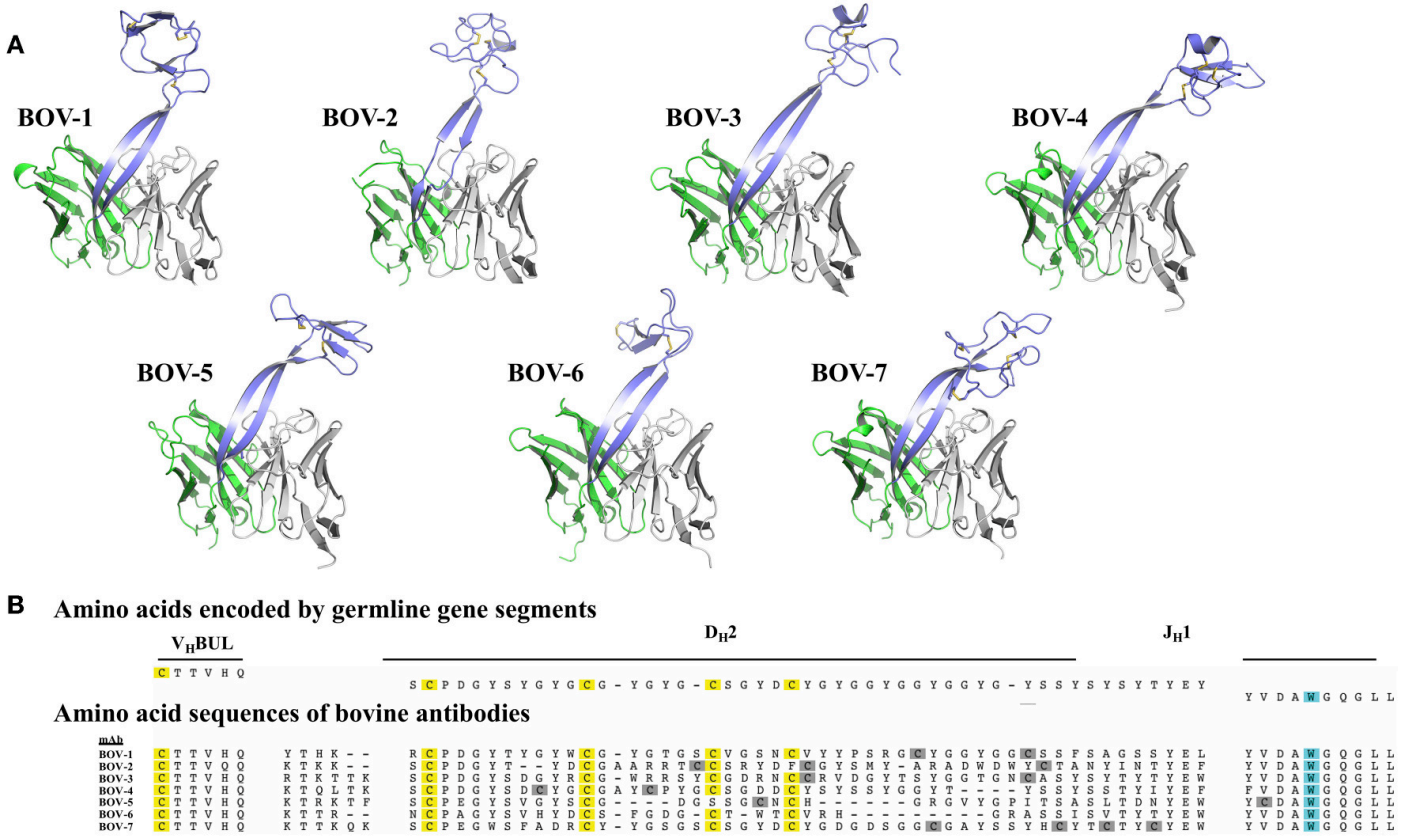
Bovine Fv crystal structures and sequence alignment of the CDRH3 regions. **(A)** Crystal structures of the Fv regions of antibodies BOV-1, BOV-2, BOV-3, BOV-4, BOV-5, BOV-6, BOV-7. Antibody light chain variable domains are colored gray, heavy chain variable domains are colored green, except that CDRH3 regions are colored light blue. **(B)** The amino acid sequences of the CDRH3s were aligned using MUSCLE (30) with sequences encoded by the V_H_BUL, D_H_2, and J_H_1 germline encoded gene segments. Cysteine (C) residues possibly from germline are highlighted in yellow, and cysteine residues possibly from mutations are highlighted in gray; the tryptophan (W) residue that marks the C-terminus of the CDRH3 region is highlighted in cyan.

Consistent with the five structures solved previously (18, 20), the 7 new CDRH3 structures also showed a long “stalk” formed by 2 anti-parallel β-strands supporting a compact, globular “knob” (Figure 1A), further establishing the stalk-knob configuration as the general structural peculiarity for the ultralong bovine CDRH3s. We noted structural diversity in both the collection of stalks and knobs.

The protein sequences of the 7 CDRH3s are highly diverse, with lengths ranging from 52 to 63 amino acids and numbers of cysteines from 4 to 8 (Figure 1B). In every case except for Bov-5 (having a free cysteine close to the C-terminal end of CDRH3, Table 1), the cysteines occurred in pairs, and every cysteine participated in a disulfide bond. The pairing patterns were diverse (Table 1). The two antibodies with 4 or 5 cysteines (BOV-5 and BOV-6) both used a [1-4, 2-3] cysteine pairing pattern. The four antibodies with 6 cysteines (BOV-1, BOV-2, BOV-3, BOV-4) used three different cysteine pairing patterns; BOV-1 and BOV-2 used [1-3, 2-5, 4-6], while BOV-3 used [1-4, 2-5, 3-6] and BOV-4 used [1-6, 2-5, 3-4] pairing patterns. The one antibody with 8 cysteines (BOV-8) used a [1-2, 3-8, 4-7, 5-6] pattern.

**Table 1:** Diverse cysteine pairing patterns in ultralong CDRH3s of bovine antibodies.

**mAb**	**CDR length (aa)**	**# of Cys**	**Cysteine pairing pattern**	**CDRH3 amino acid sequence (cysteine pairs indicated by font features)**	**Reference**
BOV-1	63	6	**1-3**, 2-5, **4-6**	TTVHQYTHKR**C**^**1**^PDGYTYGYWC^2^GYGTGS**C**^**3**^VGSN***C**^**4**^*VYYPSRGC^5^YGGYGG***C**^**6**^*SSFSAGSSYELYVDA	This study
BOV-2	61			TTVQQKTKKS**C**^**1**^PDGYTYDC^2^GAARRT**C**^**3**^***C**^**4**^*SRYDFC^5^GYSMYARADWDWY***C**^**6**^*TANYINTYEFYVDA	
BOV-3	65		**1-4**, 2-5, **3-6**	TTVHQRTKTTKS**C**^**1**^PDGYSDGYRC^2^GWRRSY***C**^**3**^*GDRN**C**^**4**^C^5^RVDGYTSYGGTGN***C**^**6**^*ASYSYTYTYEWYVDA	
BOV-4	62		**1-6**, 2-5, **3-4**	TTVHQKTQLTKS**C**^**1**^PDGYSDC^2^YG***C**^**3**^*GAY***C**^**4**^*PYGC^5^SGDD**C**^**6**^YSYSSYGGYTYSSYSSTYIYEFFVDA	
BOV-5	56	5	**1-4**, 2-3	TTVHQKTRKTFS**C**^**1**^PEGYSVGYSC^2^GDGSSGC^3^N*C*^**4**^HGRGVYGPITSASLTDNYEWYC^5^DA	
BOV-6	52	4	**1-4**, 2-3	TTVHQKTTRN**C**^**1**^PAGYSVHYDC^2^SFGDGC^3^TWT***C**^**4**^*VRHGRASSISVTYTYEWYVDA	
BOV-7	65	8	**1-2**, 3-8, **4-7**, 5-6	TTVHQKTTKQKS**C**^**1**^PEGWSFADR**C**^**2**^YYGSGSC^3^SGYD***C**^**4**^*YGDGDSGGC^5^GAYSSYHC^6^YT***C**^**7**^*TYC^8^YEWYVDA	
BLV1H12^*^	63	6	**1-4**, 2-6, 3-5	TSVHQETKKYQS**C**^**1**^PDGYRERSDC^2^SNRPAC^3^GTSD**C**^**4**^C^5^RVSVFGNC^6^LTTLPVSYSYTYNYEWHVDV	PDB: 4K3D
BLV5B8^*^	58	6	**1-3**, 2-4, 5-6	TTVHQETRKT**C**^**1**^SDGYIAVDSC^2^GRGQSDG**C**^**3**^VNDC^4^NSC^5^YYGWRNC^6^RRQPAIHSYEFHVDA	PDB: 4K3E
E03^**^	48	2	1-2	TTVHQKTLEVRSC^1^PDGSRLIGNDC^2^RNEDGDDVNYITTFDYEWYVDA	PDB: 5IJV
B11^**^	65	8	**1-4**, 2-7, **3-8**, 5-6	VTVYQKTTQKKN**C**^**1**^PDDYTEC^2^YGGA***C**^**3**^*DGTG**C**^**4**^C^5^SGSC^6^GGASAC^7^RDWWPYRSI***C**^**8**^*SSDNTYTYEFHVDA	PDB: 5IHU
A01^**^	63	6	**1-4**, 2-5, 3-6	TIVHQETSRRGPDGYSWI**C**^**1**^EC^2^SSGTYTC^3^DADN**C**^**4**^GNLC^5^PSDWQLTLHC^6^HRLDSSTYTYDWHVET	PDB: 5ILT

* From Wang et al. (18);

** *From Stanfield et al. (20)*.

*Cysteines that pair are indicated with matching plain, bold or ud font to facilitate visual recognition of the patterns of pairing in the CDRH3 amino acid sequence*.

### Diversity of CDRH3 Stalk Structures

**Figure 2:**
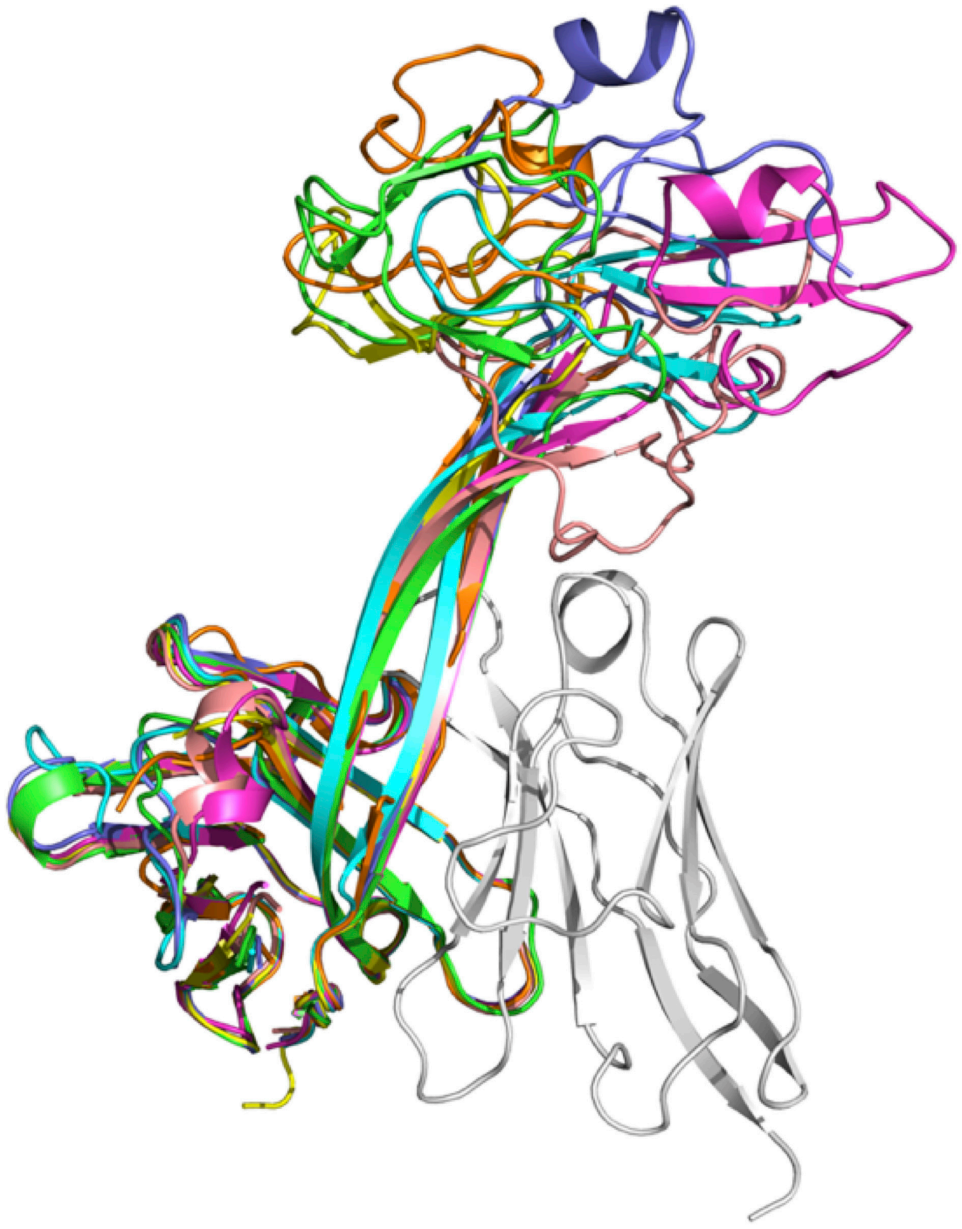
Superimposition of seven crystal structures of bovine heavy chain variable domains. Antibody BOV-1 is in green, BOV-2 in orange, BOV-5 in cyan, BOV-4 in magenta, BOV-6 in yellow, BOV-3 in light blue, and BOV-7 in pink. The light chain variable domain of antibody BOV-2 is shown in gray for reference.

The lengths of these stalks project the top of the knob domains far beyond the surface of the other heavy chain CDRs. Interestingly, not all of the stalks possess continuous anti-parallel β-strands, as shown in the stalk of the antibody BOV-2 CDRH3 (Figure 1A). The relative orientations of the stalks within the overall antibody structures are very similar, with the stalks tilting toward the light chain variable domains (Figure 2). The tilting angle differences between different CDRH3s are <7°. However, since the stalks are long, small tilting angle differences result in relatively large shifts of the relative knob positions at the distal tip of the loops. In addition, all stalks form curved β-strands, and they have different lengths due to the presence of nucleotide additions between the V_H_BUL and D_H_2 gene segments that encode an additional 4 or 6 amino acids (Figure 1B). Therefore, differences in tilting angles, stalk length, and stalk curvature work together to modulate the relative positions of the knobs displayed by each stalk.

### Diversity of Knob Structures

The knob regions exhibit very different shapes, caused by highly diverse amino acid sequences, cysteine residue positions, and disulfide bond configurations (Figures 1, 2). Many globular proteins or protein domains in nature possess hydrophobic cores, but the knob structures here all lack such a core. The compact globular folds of the knobs are maintained primarily by several disulfide bonds, a feature that is similar to the core of other small disulfide rich proteins or domains such as voltage-gated sodium channel antagonist mu-conopeptide, CnIIIC (31). We searched the PDB database using the DALI Server (32), but did not find any protein domains with significant structural homology to any of the knobs studied here.

**Figure 3:**
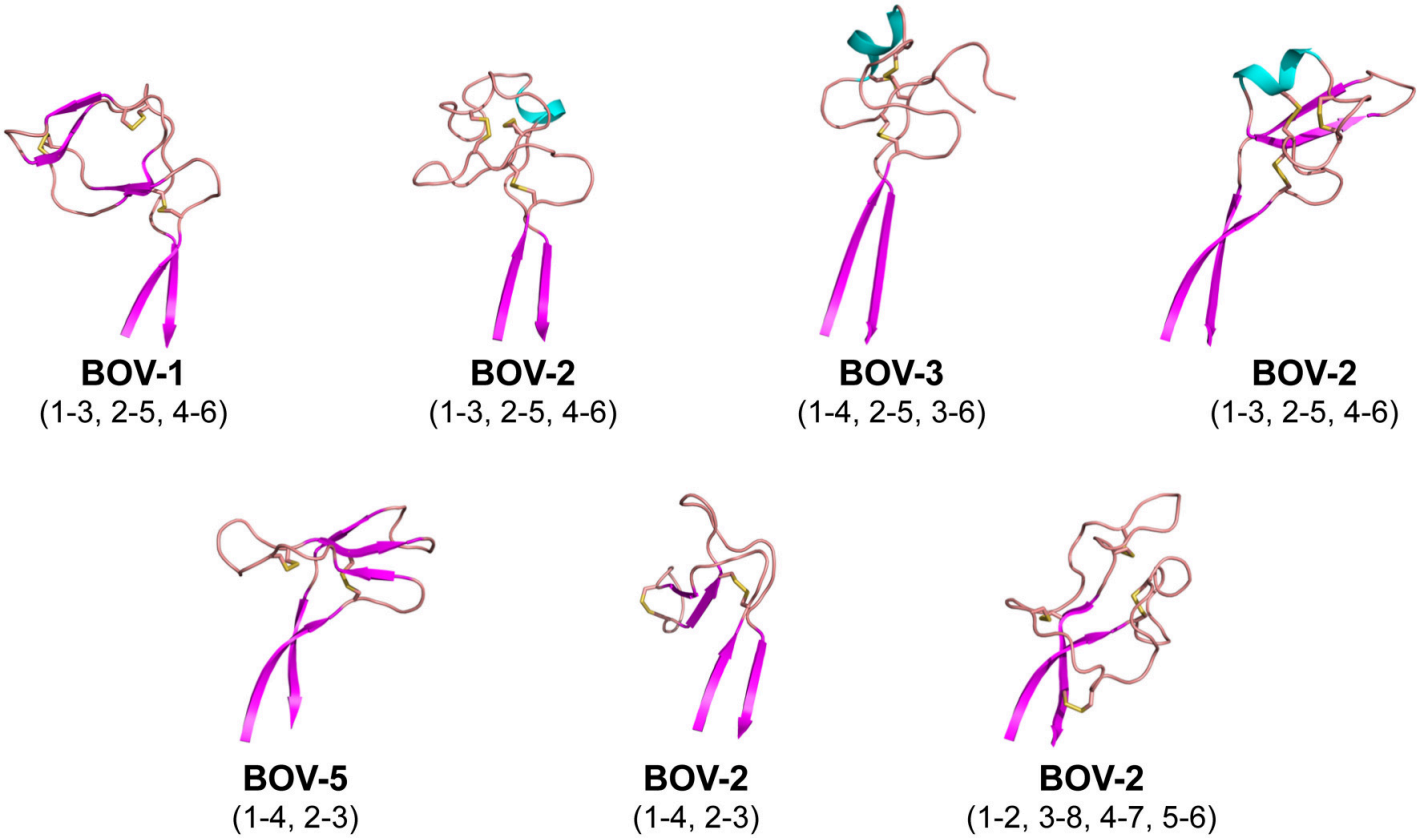
Cartoon representations of the knob structures on the distal tip of seven ultralong CDRH3s of bovine antibodies. β strands are colored magenta, α helices are colored cyan, and loops colored salmon. Sulfur atoms are shown in yellow to indicate disulfide bonds. Disulfide bond (cysteine pairing) configurations are in parentheses. Part of one loop in antibody BOV-3 was disordered in the crystal structure; this region is represented with a dashed line.

Each of the knob structures starts with the conserved sequence motif [CP(D/E/A)G(Y/W)], in which the initial cysteine residue forms the first disulfide bond with another cysteine residue in the knob, and the motif forms a type I β turn (Figure 3). Six of the seven new knobs studied here have general structural features similar to those of the five bovine knobs previously described (18, 20). The exception, the knob of antibody BOV-7, will be discussed separately below. Two or three anti-parallel peptide segments make β-strands or β-strand-like backbone conformations in the knobs, and the first disulfide bond forms between the conserved cysteine residue and another cysteine residue within the second segment. A typical example of this feature is the knob of antibody BOV-5 (Figure 3). Depending on number and locations of disulfide bonds and lengths and sequences of segments between these disulfide bonds, some knobs have additional structural elements packed against the three antiparallel segments. The knob of antibody BOV-1 has 2 additional anti-parallel β-strands, extending from the second and the third of the three antiparallel segments. For the antibody BOV-2, a one-turn α-helix and a long loop is packed on top of the knob, again connecting the second and the third of the three antiparallel segments. In contrast, in the knobs of antibodies BOV-4 and BOV-3, a two-turn α-helix and a long loop extend from the first and the second of the three antiparallel segments. These four knobs could be described as two-layered structures with the three antiparallel segments as the first layer, and the additional structural elements packed against the first layer as the second layer. The knobs of antibody BOV-5 and BOV-6 form one-layered structures without the second layer seen in antibodies BOV-1–BOV-4. BOV-5 and BOV-6 have relatively short CDRH3s, which might be due to deletion mutations in the region encoded by the D_H_2 segments (Figure 1A). D_H_2 deletion mutations have been observed in bovine ultralong CDRH3s, contributing to diversification of bovine antibody repertoires (33). Interestingly, deletion mutations in the region encoded by the D_H_2 segment in BOV-5 and BOV-6 reduce “two-layered” knob structures to “single-layered” ones. This instance of structural common structural rearrangements associated with D gene region deletions is interesting. However, it is difficult with a limited number of such examples to suggest a generalized “rule” for the structural effects of deletion mutations.

**Figure 4:**
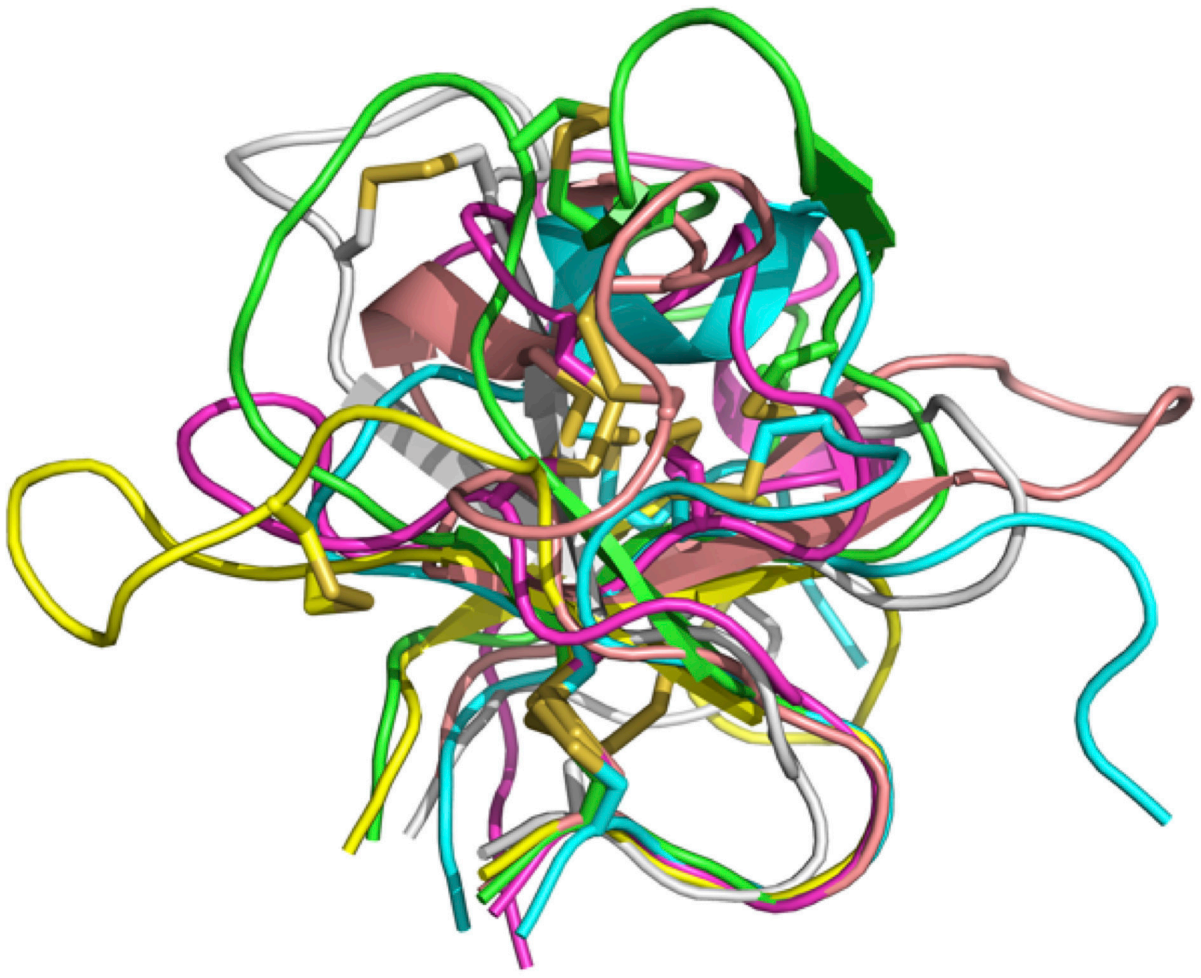
Overlay of six bovine knob structures. The knob for antibody BOV-1 is colored in green, BOV-2 in magenta, BOV-3 in cyan, BOV-4 in salmon, BOV-5 in yellow, and BOV-6 in gray. Cysteine residues are shown in stick representation.

In sum, despite some general similarities, each of the knobs exhibits a unique conformation, reflecting the diversity of sequences and disulfide configurations (Figure 4).

### An Unusual Knob/Stalk Interaction

Interestingly, the antibody BOV-7 knob has very different structural features from the other knobs. This knob consists of five loops separated by 4 disulfide bridges without any α-helical or β-strand secondary structure elements, and four of the five loops are more than 6 residues long. Two of the four disulfide bonds are formed between cysteine residues in the knob region and cysteine residues in the stalk, resulting in three loops that are partially buried in the stalk. Therefore, the antibody BOV-7 knob, being structurally intertwined with the stalk, shows distinct structural features different from any other bovine CDRH3 knob described.

### Structural Flexibility of Ultralong CDRH3s

**Figure 5:**
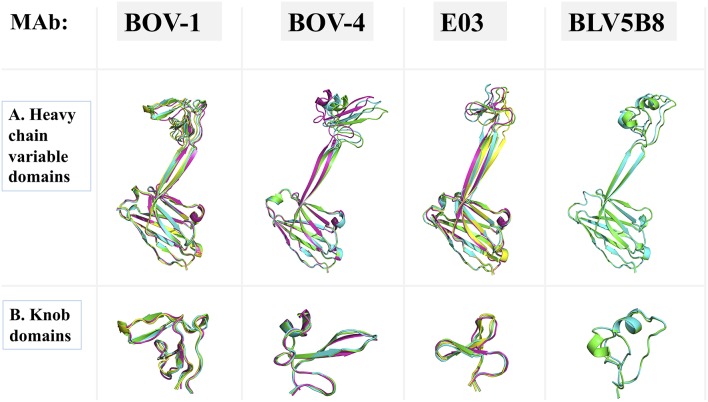
Superimpositions of antibody domains of Fab copies within one asymmetric unit. Fab structures for antibodies BOV-1 and BOV-4 are from this study. BLV5B8 (PDB ID: 4K3E) and E03 (PDB ID: 5IJV) are from Koti et al. (17) and Wang et al. (18), respectively. Copies within one asymmetric unit are represented in cartoon with different colors. **(A)** Heavy chain variable domains. **(B)** Knob domains.

In the crystal structures of antibodies BOV-6 and BOV-1, there are 4 and 8 copies of Fab molecules in one asymmetric unit, respectively. However, electron density associated with 4 or 3 knob regions of antibodies BOV-1 or BOV-6 is mostly or totally absent, suggesting that these ultralong CDRH3s are highly flexible. The flexibility may originate from characteristics of the particular stalks and/or knobs used. To examine the extent of this flexibility, we superimposed the heavy chain variable domains of each Fab molecule in the asymmetric unit for antibodies BOV-1 or BOV-4 in one asymmetric unit (Figure 5A). The stalk orientations of stalks of Fab copies within an asymmetric unit for antibodies BOV-1 or BOV-4 significantly differ from the rest of heavy chain variable domains, which superimpose on one another. The twisting angles between the two strands of stalks are also different among copies of the Fabs within one asymmetric unit, suggesting that the CDRH3 stalks could undergo tilting and twisting motions due to their inherent flexibility. We also compared the heavy chain variable domains of copies of the previously described Fab E03 (20) and Fab BLV5B8 (18) within an asymmetric unit (Figure 5A). Similar to those of antibodies BOV-1 and BOV-4, the Fab E03 stalk shows tilting and twisting differences among different copies but to a larger extent. In contrast, the Fab BLV5B8 stalk shows little tilting and twisting, but the superimposition suggests a rigid-body rotation of the knob hinged at the junction between the knob and the stalk (Figure 5A). The superimposition of knobs on these Fabs within an individual asymmetric unit indicates that there are only small structural variations in knobs, suggesting that the knobs are structurally stable (Figure 5B). The structural flexibility of the stalks, and the hinge regions between the stalks and knobs, explains the lack of the electron density in some knob regions of these Fabs. After examining all the crystal contacts of these bovine Fab crystal structures, we found that all of the knob regions with traceable electron density form crystal contacts with other structural elements in the protein crystals, restricting the motions of the knob regions to make these regions visible. This finding further supports the idea of significant flexibility in the stalk and the hinge regions.

## Discussion

Here we report 7 diverse structures of ultralong bovine CDRH3s, including a novel mAb with stabilizing interactions between the knob and stalk. The results document very high structural diversity of bovine CDRH3s in the crystal structures solved, especially in the knob regions. In addition, the knob structure of one ultralong CDRH3 BOV-7 shows a novel fold different from others, suggesting that bovine CDRH3 repertoire might construct additional knob patterns to what has been recognized so far. The knob structures solved in this study exhibit unique features, based on the high sequence variety of bovine ultralong CDRH3s, and further elucidate the structural diversity of small disulfide-rich protein domains. These structures, combined with the five bovine Fab structures solved previously (18, 20), suggests that the organization of ultralong CDRH3s into well-defined three-dimensional structures mainly via disulfide bridges may allow bovine repertoires to achieve a similar range of antigen recognition to that of humans using a smaller overall number of gene segments but a locally higher degree of sequence and structural diversity in CDRH3s. Since there are no deep comparative studies on the antibody repertoire complexity between human and cattle, we do not know which type of repertoire is more genetically diverse. Likely, the ultralong CDRH3 configuration, incorporating globular knobs with high levels of loop diversity, allow cattle to achieve an enhanced level of structural diversity in the antibody repertoire with a smaller number of variable gene segments (23).

The protruding CDRH3 of bovine antibodies with ultralong loop structures may provide these bovine antibodies with a special advantage to bind to epitopes that are not easily accessible to common antibodies with flatter paratope features. Some evidence supports that the knobs of these antibodies are sufficient for antigen binding (18, 22). If so, then the stalk region may serve essentially as a display scaffold to deliver the knob domain, which functions as a mini-antibody or effector domain that mediates molecular recognition of antigen. Considering this possibility, the functional definition of the paratope in bovine antibodies with ultralong CDRH3s might be limited to the knob domain. These knobs displayed on ultralong CDRH3s may have a structural advantage that allows them to interact with protein antigens that are obscured by post-translational modifications of foreign antigens. For example, long CDRH3s in rare human mAbs facilitate penetration of the glycan shield of the HIV envelope glycoprotein to allow interaction with amino acids in the envelope that are otherwise inaccessible to recognition by antibody paratopes (22). The CDRH3s described here could be used in the future for protein engineering purposes, when small globular protein scaffolds are needed.

The structural evidence in this study suggests that the stalk and the knob/stalk hinge region are highly flexible, and this flexibility might in some cases facilitate the recognition processes between antibodies and antigens. The flexibility of the stalk and the hinge regions may have functional consequences for these bovine antibodies. Flexible stalks and the hinges may modulate the thermodynamics and/or kinetics of antibody binding to the corresponding antigens, even if those domains are not involved directly in binding. Thus, it may be kinetically beneficial for these ultralong CDRH3s to have flexible stalks and stalk-knob junctions in order to recognize epitopes in deep cavities or crevices. The flexible knob/stalk configuration may enhance access of the knob to occult epitopes on foreign antigens that otherwise could not be recognized. On the other hand, preconfiguring the antigen-bound conformation of antibody long CDRH3s by reducing the flexibility of the loops can enhance affinity and (34). Further studies are needed to address these possibilities.

## Data Availability

Atomic coordinates and structure factors for the crystal structures have been deposited in the Protein Data Bank with the accession codes as follows: BOV-1 PDB ID 6e8v, BOV-2 PDB ID 6e9G, BOV-3 PDB ID 6e9h, BOV-4 PDB ID 6e9i, BOV-5 PDB ID 6e9k, BOV-6 PDB ID 6e9q, BOV-7 PDB ID 6e9u.

## Author Contributions

JD and JC conceived and designed the research. PL and TS provided the antibody sequences. JF analyzed sequences. JD expressed recombinant antibodies and determined the X-ray structure of the antibodies. JD and JC wrote the manuscript. All authors edited and approved the final manuscript.

### Conflict of Interest Statement

JC is a consultant for Sanofi, is on the Scientific Advisory Boards of CompuVax and Meissa Vaccines, is a recipient of previous unrelated research grants from Moderna and Sanofi and is a founder of IDBiologics. The remaining authors declare that the research was conducted in the absence of any commercial or financial relationships that could be construed as a potential conflict of interest.

## References

[1] Ratcliffe MJ Jacobsen KA. Rearrangement of immunoglobulin genes in chicken B cell development. Semin Immunol. (1994) 6:175–84. 10.1006/smim.1994.1023 7948957

[2] Wu L Oficjalska K Lambert M Fennell BJ Darmanin-Sheehan A Ni Shuilleabhain D. Fundamental characteristics of the immunoglobulin VH repertoire of chickens in comparison with those of humans, mice, and camelids. J Immunol. (2012) 188:322–33. 10.4049/jimmunol.1102466 22131336

[3] Shih HH Tu C Cao W Klein A Ramsey R Fennell BJ. An ultra-specific avian antibody to phosphorylated tau protein reveals a unique mechanism for phosphoepitope recognition. J Biol Chem. (2012) 287:44425–34. 10.1074/jbc.M112.415935 23148212 PMC3531756

[4] Conroy PJ Law RH Gilgunn S Hearty S Caradoc-Davies TT Lloyd G. Reconciling the structural attributes of avian antibodies. J Biol Chem. (2014) 289:15384–92. 10.1074/jbc.M114.562470 24737329 PMC4140895

[5] Weber J Peng H Rader C. From rabbit antibody repertoires to rabbit monoclonal antibodies. Exp Mol Med. (2017) 49:e305. 10.1038/emm.2017.23 28336958 PMC5382564

[6] Nguyen VK Hamers R Wyns L Muyldermans S. Camel heavy-chain antibodies: diverse germline V(H)H and specific mechanisms enlarge the antigen-binding repertoire. EMBO J. (2000) 19:921–30. 10.1093/emboj/19.5.921 10698934 PMC305632

[7] Johansson J Aveskogh M Munday B Hellman L. Heavy chain V region diversity in the duck-billed platypus (*Ornithorhynchus anatinus*): long and highly variable complementarity-determining region 3 compensates for limited germline diversity. J Immunol. (2002) 168:5155–62. 10.4049/jimmunol.168.10.5155 11994470

[8] Walther S Czerny CP Diesterbeck US Exceptionally long CDR3H are not isotype restricted in bovine immunoglobulins. PLoS ONE. (2013) 8:e64234 10.1371/journal.pone.0064234 23717573 PMC3661452

[9] Walther S Tietze M Czerny CP Konig S Diesterbeck US. Development of a bioinformatics framework for the detection of gene conversion and the analysis of combinatorial diversity in immunoglobulin heavy chains in four cattle breeds. PLoS ONE. (2016) 11:e0164567. 10.1371/journal.pone.0164567 27828971 PMC5102495

[10] Saini SS Kaushik A. Extensive CDR3H length heterogeneity exists in bovine foetal VDJ rearrangements. Scand J Immunol. (2002) 55:140–8. 10.1046/j.1365-3083.2002.01028.x 11896930

[11] Saini SS Kaushik A. Origin of bovine IgM structural variants. Mol Immunol. (2001) 38:389–96. 10.1016/S0161-5890(01)00063-3 11684295

[12] Saini SS Farrugia W Ramsland PA Kaushik AK. Bovine IgM antibodies with exceptionally long complementarity-determining region 3 of the heavy chain share unique structural properties conferring restricted VH + Vlambda pairings. Int Immunol. (2003) 15:845–53. 10.1093/intimm/dxg083 12807823

[13] Saini SS Allore B Jacobs RM Kaushik A. Exceptionally long CDR3H region with multiple cysteine residues in functional bovine IgM antibodies. Eur J Immunol. (1999) 29:2420–6. 10.1002/(SICI)1521-4141(199908)29:08<2420::AID-IMMU2420>3.0.CO;2-A 10458755

[14] Kaushik AK Kehrli ME Jr Kurtz A Ng S Koti M Shojaei F Somatic hypermutations and isotype restricted exceptionally long CDR3H contribute to antibody diversification in cattle. Vet Immunol Immunopathol. (2009) 127:106–13. 10.1016/j.vetimm.2008.09.024 19012969

[15] Kaushik A Shojaei F Saini SS. Novel insight into antibody diversification from cattle. Vet Immunol Immunopathol. (2002) 87:347–50. 10.1016/S0165-2427(02)00063-6 12072257

[16] Shi B Ma L He X Wang X Wang P Zhou L. Comparative analysis of human and mouse immunoglobulin variable heavy regions from IMGT/LIGM-DB with IMGT/HighV-QUEST. Theor Biol Med Model. (2014) 11:30. 10.1186/1742-4682-11-30 24992938 PMC4085081

[17] Koti M Kataeva G Kaushik AK. Novel atypical nucleotide insertions specifically at VH-DH junction generate exceptionally long CDR3H in cattle antibodies. Mol Immunol. (2010) 47:2119–28. 10.1016/j.molimm.2010.02.014 20435350

[18] Wang F Ekiert DC Ahmad I Yu W Zhang Y Bazirgan O. Reshaping antibody diversity. Cell. (2013) 153:1379–93. 10.1016/j.cell.2013.04.049 23746848 PMC4007204

[19] Cheek S Krishna SS Grishin NV. Structural classification of small, disulfide-rich protein domains. J Mol Biol. (2006) 359:215–37. 10.1016/j.jmb.2006.03.017 16618491

[20] Stanfield RL Wilson IA Smider VV. Conservation and diversity in the ultralong third heavy-chain complementarity-determining region of bovine antibodies. Sci Immunol. (2016) 1:aaf7962. 10.1126/sciimmunol.aaf7962 27574710 PMC5000368

[21] Doria-Rose NA Schramm CA Gorman J Moore PL Bhiman JN DeKosky BJ. Developmental pathway for potent V1V2-directed HIV-neutralizing antibodies. Nature. (2014) 509:55–62. 10.1038/nature13036 24590074 PMC4395007

[22] Sok D Le KM Vadnais M Saye-Francisco KL Jardine JG Torres JL. Rapid elicitation of broadly neutralizing antibodies to HIV by immunization in cows. Nature. (2017) 548:108–11. 10.1038/nature23301 28726771 PMC5812458

[23] Larsen PA Smith TP. Application of circular consensus sequencing and network analysis to characterize the bovine IgG repertoire. BMC Immunol. (2012) 13:52. 10.1186/1471-2172-13-52 22978666 PMC3500647

[24] Kabsch W Xds. Acta Crystallogr D Biol Crystallogr. (2010) 66(Pt 2):125–32. 10.1107/S0907444909047337 20124692 PMC2815665

[25] Winn MD Ballard CC Cowtan KD Dodson EJ Emsley P Evans PR. Overview of the CCP4 suite and current developments. Acta Crystallogr D. (2011) 67:235–42. 10.1107/S0907444910045749 21460441 PMC3069738

[26] Mccoy AJ Grosse-Kunstleve RW Adams PD Winn MD Storoni LC Read RJ. phaser crystallographic software. J Appl Crystallogr. (2007) 40:658–74. 10.1107/S0021889807021206 19461840 PMC2483472

[27] Adams PD Afonine PV Bunkoczi G Chen VB Davis IW Echols N. PHENIX: a comprehensive python-based system for macromolecular structure solution. Acta Crystallogr D. (2010) 66:213–21. 10.1107/S0907444909052925 20124702 PMC2815670

[28] Emsley P Cowtan K. Coot: model-building tools for molecular graphics. Acta Crystallogr D. (2004) 60:2126–32. 10.1107/S0907444904019158 15572765

[29] Schrodinger LLC The PyMOL Molecular Graphics System, Version 1.8. New York, NY (2015).

[30] Edgar RC. MUSCLE: multiple sequence alignment with high accuracy and high throughput. Nucleic Acids Res. (2004) 32:1792–7. 10.1093/nar/gkh340 15034147 PMC390337

[31] Favreau P Benoit E Hocking HG Carlier L D' hoedt D Leipold E. A novel micro-conopeptide, CnIIIC, exerts potent and preferential inhibition of NaV1.2/1.4 channels and blocks neuronal nicotinic acetylcholine receptors. Br J Pharmacol. (2012) 166:1654–68. 10.1111/j.1476-5381.2012.01837.x 22229737 PMC3419909

[32] Holm L Rosenstrom P. Dali server: conservation mapping in 3D. Nucleic Acids Res. (2010) 38:W545–9. 10.1093/nar/gkq366 20457744 PMC2896194

[33] Deiss TC Vadnais M Wang F Chen PL Torkamani A Mwangi W. Immunogenetic factors driving formation of ultralong VH CDR3 in Bos taurus antibodies. Cell Mol Immunol. (2019) 16:53–64. 10.1038/cmi.2017.117 29200193 PMC6318308

[34] Willis JR Sapparapu G Murrell S Julien JP Singh V King HG. Redesigned HIV antibodies exhibit enhanced neutralizing potency and breadth. J Clin Invest. (2015) 125:2523–31. 10.1172/JCI80693 25985274 PMC4497764

