# Supplementary material for: Structural Diversity of Ultralong CDRH3s in Seven Bovine Antibody Heavy Chains
Source: Front Immunol. 2019 Mar 22;10:558. doi: 10.3389/fimmu.2019.00558 (PMC6440498; doi:10.3389/fimmu.2019.00558)
Supplement: Supplementary file 1 [file Table_1.DOCX]

Supplementary Material

**Structural Diversity of Ultralong CDRH3s in Seven Bovine Antibody Heavy Chains**

| **Table S1. Crystallization and cryo-protection conditions of bovine Fabs** | | |
| --- | --- | --- |
| **Fab** | **Crystallization condition** | **Cryo-protection condition** |
| BOV-4 | 30% PEG 1000, 0.1 M sodium malonate pH 8.0, 0.1 M Tris-HCl pH 8.0 | 36.5% PEG 1000, 0.1 sodium malonate pH 8.0, 0.1 M Tris-HCl pH 8.0 |
| BOV-1 | 14-18% PEG 3350, 0.1 M citric acid pH 4.0-4.5, 0.1 M sodium citrate pH 4.5 | 25% glycerol, 14-18% PEG 3350, 0.1 M citric acid pH 4.0-4.5, 0.1 M sodium citrate pH 4.5 |
| BOV-2 | 17% PEG 8000, 10% PEG 200, 0.1 M Bis-Tris propane pH 9.0-9.5 | 17% PEG 8000, 30% PEG 200, 0.1 M Bis-Tris propane pH 9.0-9.5 |
| BOV-3 | 30% PEG 1000, 0.1 M sodium malonate pH 8.0, 0.1 M Tris-HCl pH 8.0 | 40% PEG 1000, 0.1 M sodium malonate pH 8.0, 0.1 M Tris-HCl pH 8.0 |
| BOV-7 | 7% PEG 8000, 14% 2-propanol, 0.1 M Tris-HCl pH 7.5 | 7% PEG 8000, 25% 2-propanol, 0.1 M Tris-HCl pH 7.5 |
| BOV-5 | 25% PEG 4000, 6% tacsimate, 0.1 M MES pH 6.0 | 23% glycerol, 25% PEG 4000, 6% tacsimate, 0.1 M MES pH 6.0 |
| BOV-6 | 18% PEG 8000, 0.1 M HEPES pH 7.5 | 23% glycerol, 18% PEG 8000, 0.1 M HEPES pH 7.5 |

Jinhui Dong*, Jessica A. Finn, Peter A. Larsen, Timothy P. L. Smith, and James E. Crowe, Jr.

*** Correspondence:** James E. Crowe, Jr.: james.crowe@vanderbilt.edu

# Supplementary Tables

| Data collection |  |  |  |  |  |  |  |
| --- | --- | --- | --- | --- | --- | --- | --- |
| Crystal | Bov-1 | Bov-2 | Bov-3 | Bov-4 | Bov-5 | Bov-6 | Bov-7 |
| PDB ID | 6E8V | 6E9G | 6E9H | 6E9I | 6E9K | 6E9Q | 6E9U |
| Wave Length (Å) | 0.97856 | 0.97856 | 0.97872 | 0.97856 | 0.97856 | 0.97856 | 0.97856 |
| Space group | P4_2_2_1_2 | C121 | P2_1_2_1_2_1_ | P3_2_21 | P1211 | P2_1_2_1_2_1_ | P12_1_1 |
| Unit cell dimensions |  |  |  |  |  |  |  |
| a, b, c (Å) | 308.2, 308.2, 133.8 | 82.4, 71.9, 88.0 | 51.3, 71.2, 119.8 | 148.5, 148.5, 165.4 | 45.0, 70.8, 73.9 | 64.0, 72.7, 452.9 | 50.3, 79.3, 62.2 |
| α, β, γ | 90.0, 90.0, 90.0 | 90.0, 96.56, 90.0 | 90.0, 90.0, 90.0 | 90.0, 90.0, 120.0 | 90.0, 107.1, 90.0 | 90.0, 90.0, 90.0 | 90.0, 95.2, 90.0 |
| Resolution (Å) | 49.83 – 3.79 | 47.59 – 2.90 | 47.12 – 2.00 | 41.87 – 2.50 | 43.03 – 2.19 | 48.83 – 3.40 | 48.79 – 2.29 |
| Unique reflections | 64320 (9257) | 11347 (1586) | 30426 (4354) | 66205 (9588) | 21489 (2221) | 30383 (4372) | 21713 (3106) |
| Redundancy | 7.5 (7.6) | 3.7 (3.6) | 7.3 (7.4) | 6.3 (6.3) | 3.6 (2.8) | 7.3 (7.5) | 3.8 (3.7) |
| Completeness (%) | 100.0 (100.0) | 99.6 (97.0) | 100.0 (100.0) | 90.1 (90.3) | 94.0 (66.9) | 100.0 (100.0) | 99.5 (98.5) |
| R_merge_ (%) | 16.6 (65.0) | 5.9 (23.0) | 5.4 (38.6) | 5.6 (45.6) | 3.7 (42.8) | 10.9 (31.0) | 4.9 (33.3) |
| I/σ(I) | 10.6 (3.5) | 13.6 (4.4) | 18.6 (4.9) | 20.3 (4.2) | 13.5 (2.1) | 11.8 (4.9) | 15.4 (3.6) |
| Refinement statitics |  |  |  |  |  |  |  |
| R_factor_ | 21.83 | 22.04 | 21.70 | 20.24 | 20.42 | 25.40 | 25.65 |
| R_free_ | 27.45 | 26.31 | 25.56 | 24.31 | 26.41 | 31.07 | 21.38 |
| R.m.s.d. (bond) (Å) | 0.0040 | 0.0020 | 0.0017 | 0.0029 | 0.0028 | 0.0017 | 0.0021 |
| R.m.s.d. (angle) (deg) | 0.654 | 0.581 | 0.493 | 0.597 | 0.562 | 0.505 | 0.554 |
| Ramachandran plot |  |  |  |  |  |  |  |
| Favored (%) | 93.62 | 93.54 | 97.60 | 97.59 | 98.00 | 93.63 | 97.03 |
| Allowed (%) | 6.01 | 6.24 | 2.40 | 2.41 | 2.00 | 6.07 | 2.76 |
| Outliers (%) | 0.37 | 0.22 | 0.00 | 0.00 | 0.00 | 0.30 | 0.21 |

**Table S2.** Data collection and refinement statistics for the crystals of bovine antibodies with ultralong CDRH3s
